# Undergraduate ultrasound training: prospective comparison of two different peer assisted course models on national standards

**DOI:** 10.1186/s12909-023-04511-x

**Published:** 2023-07-17

**Authors:** Johannes Weimer, Anna Dionysopoulou, Kai-Uwe Strelow, Holger Buggenhagen, Julia Weinmann-Menke, Klaus Dirks, Andreas Weimer, Julian Künzel, Norbert Börner, Michael Ludwig, Yang Yang, Liv Lorenz, Carlotta Ille, Lukas Müller

**Affiliations:** 1 Rudolf Frey Learning Clinic, University Medical Center of the Johannes Gutenberg University Mainz, Mainz, Germany; 2 Department of obstetrics and Gynecology, University Medical Center of the Johannes Gutenberg University Mainz, Mainz, Germany; 3 Department of Medicine, University Medical Center of the Johannes Gutenberg University Mainz, Mainz, Germany; 4 Department of General Internal Medicine and Geriatrics, Rems-Murr-Klinikum, Winnenden, Germany; 5 Center of Orthopedics, Trauma Surgery, and Spinal Cord Injury, Heidelberg University Hospital Heidelberg, Heidelberg, Germany; 6 Department of Otorhinolaryngology, Head and Neck Surgery, University Hospital Regensburg, Regensburg, Germany; 7 Gastroenterological Medical Group Offices, MED Specialist Center Mainz, Mainz, Germany; 8 Department of Internal Medicine I, Hospital of the German Armed Forces Berlin, Berlin, Germany; 9 Department of Diagnostic and Interventional Radiology, University Medical Center of the Johannes Gutenberg University Mainz, Mainz, Germany; 10 Department of Radiation Oncology and Radiotherapy, University Medical Center of the Johannes Gutenberg University Mainz, Mainz, Germany

**Keywords:** Education, Ultrasound curriculum, Peer assisted learning, Course models, Undergraduate training, Curriculum developement

## Abstract

**Background:**

A thorough knowledge of sonography is essential in clinical practice. Therefore, sonography training is increasingly incorporated into the medical school curriculum, entailing different course models. The question arises which model is most effective to convey sustained sonographic skills.

**Methods:**

Two different peer-assisted learning (PAL) sonography course models were developed as part of a clinical prospective study. The course content was based on the national resident curriculum of the German Society for Ultrasound in Medicine (DEGUM). Model A consists of a 10-week course and model B of a two-day compact course. Each model entailed 20 teaching units (TU). A script was used to prepare for each unit. Two modified OSCE exams of the ultrasound skills (max = 50 points per exam) were performed during the last teaching unit to assess the competence level. For subjective self-assessment and model evaluation, a questionnaire with a 7-point Likert scale was employed.

**Results:**

A total of 888 students of the 3rd year participated as part of a voluntary elective in the study (744 in model A and 144 in model B). In the exams, participants in model A (median 43 points) scored significantly higher than those in model B (median 39; p < 0.01). Participants in model A (mean 1.71 points) obtained significantly higher mean competency gain scores in subject knowledge than model B (mean 1.43 points; p < 0.01) participants. All participants were satisfied with the course concept (A: mean 1.68 vs. B: mean 1.78 points; p = 0.05), the teaching materials (A: mean 1.81 vs. B: mean 1.69 points; p = 0.52), and the tutor’s didactic skills (A: mean 1.24 vs. B: mean 1.15 points; p < 0.05).

**Conclusion:**

These results suggest that sonography-specific competency may be obtained through different course models, with a model stretching over several weeks leading to a higher competence level. Further research should assess the long-term retention of the skills obtained in different models.

**Supplementary Information:**

The online version contains supplementary material available at 10.1186/s12909-023-04511-x.

## Background

The clinical use and significance of sonography have been increasing in recent decades, with this technique representing the most frequently used imaging procedure in the extended clinical examination in Germany [1, 2]. Sonography is a standard diagnostic tool in many medical specialties and is firmly anchored in clinical examination protocols [3]. Therefore, physicians should gain proficiency in this imaging technique at an early stage of their career to confidently apply it in the overall clinical context [1].

Training sonography in Germany is determined by the continuing education catalogs and the specifications of nationally recognized institutions and is supported by professional societies. In medical school, sonography training is administered by the respective faculties and is guided by the specifications of the examination regulations and national recommendations [4]. Based on the large number of scientific publications on this topic, it can be observed that sonography training is both nationally and internationally increasingly integrated into medical school curricula, and awareness for the importance of this diagnostic tool has been raised [5–12]. The defined learning objectives of extracurricular and curricular sonography training models entail the acquisition of skills in abdominal and emergency sonography, as well as in other specific subspecialties such as echocardiography, sonography in the head and neck region, or sonography in the musculoskeletal region. Different course models are used to train these skills, including courses spanning over an entire semester or several semesters and compact course models over a few days. In the preclinical semesters, sonography training focusses on providing an increased knowledge of anatomy and physiology [12–14], while in the clinical semesters, this knowledge is extended towards a sustained sonopathological competency [12, 15, 16]. Many training curricula rely on the use of peer tutors or peer-assisted learning (PAL) [17–19] and employ both theoretical and practical examination and evaluation formats to assess the learning process [20].

Nevertheless, many educational institutions still feel reserved when it comes to integrating structured sonography training into their curricula. Common reasons for this reluctancy are the lack of teaching time slots, the cost of sonography equipment, the lack of sufficiently trained teachers, and the lack of concepts for an efficient implementation [21]. Despite national learning objective catalogs, practical ultrasound training is conducted very heterogeneously at German universities. Currently, there is no standardized curriculum for student ultrasound teaching that is used across universities [6].

The multitude of published sonography training curricula and recommendations of international professional societies demonstrate the importance of incorporating this imaging technique into the medical school curriculum, but they also reveal the heterogeneity of the implementation approaches [6–8, 21–25]. A subjective and objective gain in sonography competency after completion of ultrasound training formats has been observed in numerous studies [26–29]. Mainly either compact formats or multi-week formats are considered. To our knowledge, no study has been conducted to date to compare different abdomen-specific ultrasound training models in terms of skill development for medical students. Therefore, the aim of the present study was to compare two different sonography course models (model A = 10-week course and model B = two-day compact course) and determine which of these models is more effective in building sonography skills. Our hypothesis was that a compact course format would build similar competencies within sonography education compared with a multi-week format. The resource requirements such as teachers and equipment, the intricacies of teaching during a pandemic as well as the educational materials are considered and discussed in this context.

## Methods

This study was conducted prospectively as an observational trial at the Rudolf-Frey Learning Clinic, I. Department of Medicine and Department of Diagnostic and Interventional Radiology, University Medical Center Mainz. It was performed in accordance with the “Strengthening the Reporting of Observational Studies in Epidemiology (STROBE) Statement: guidelines for reporting observational studies” [30]. Passing the 1st state exam and at least 80% participation in the entire course, including exams, were defined as inclusion criteria.

### Development of the course models

**Fig. 1:**
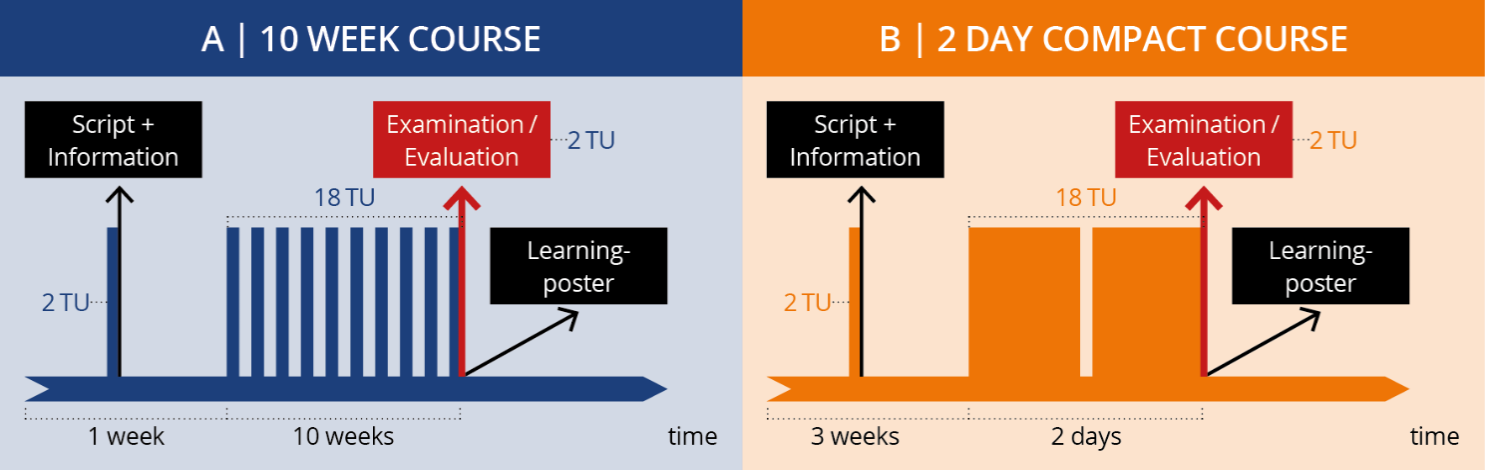
Comparison of course models A (10-week course) and B (2-day compact course)

Two sonography course models (A and B) were developed based on current national resident course curricula of the German Society for Ultrasound in Medicine (DEGUM), comparable peer-to-peer concepts, and recommendations of other professional societies [9, 15, 16, 19, 24, 31–33]. The course model A was changed due to the Corona pandemic and the study aimed to evaluate whether the new model B could achieve the same practical competencies as the previous model, which has been implemented for several semesters. Each model was composed of 20 teaching units (TU) (Fig. 1) with an emphasis on abdominal sonography (including pelvic organs) (Supplementary 1). The distribution and sequence of the different components and supplementary modules were slightly modified for didactic reasons. Medical students of the 3rd year after completion of the first state examination were included as participants. All participants completed the same practice time with the ultrasound device (approx. 120 min per person).

Participants in course model A completed a 10-week course with one session (2 TU) per week equaling 90 min. The participants received a script for course preparation. The participant-tutor-ratio was 5:1. Each session included a short review of the theoretical principles and a discussion of common pathologies in form of PowerPoint presentations. In the last session, the participants completed an ultrasound exam (two exams per person) and an evaluation. For course follow-up, the participants received an educational poster with the most important sectional planes, measurement points, and pathologies.

Course model B consisted of a 2-day compact course with the same preparation and follow-up materials as provided in course model A. The teaching content and practical exercises in course model B were conveyed according to the following principle: First, previously recorded videos on the same PowerPoint slides used in course model A were shown to the participants to repeat the theoretical principles. Subsequently, half of the participants completed practical exercises with the device with a participant-tutor ratio of 3:1. The other half of the participants received a lecture on the theory of the most common pathologies using the same PowerPoint slides as in course model A. The subgroups then switched to practice and theory, so that each participant in Group B had the same amount of time to complete both the theory and practice portions. At the end of the second course day, the participants completed the same exams and an evaluation as participants in course A. Total hands on time was calculated over the full course per participant. Several courses were held each semester.

The development and release of the script for course preparation, including high-resolution ultrasound images, were carried out in cooperation with ultrasound experts (DEGUM stage II + III), cooperation partners from various disciplines as well as didactics. The content was not fundamentally changed during the study period (except for spelling and formatting).

The script was organized in different modules according to previously defined learning objectives and included explanations on the sonographic examination procedures, imaging sections for orientation, and details on the most common pathologies. In addition, the tutors used training charts during the course to convey and visualize the content to be taught. The trained peer tutors and participants rotated during the respective course sequences so that the participants would get to know as many different lecturers as possible. The two models are shown in full extent in Supplementary Table 2.

In order to maintain objectivity and the quality of the examination, the peer tutors not only underwent a general tutor training program, but also additional specific didactic training for their use in the study. The training consisted of a multi-stage process that included participation in a DEGUM-certified ultrasound course, internal technical and pedagogical training (30 hours), and shadowing in the ultrasound laboratory (with the performance of at least 100 independent examinations). Acquisition of competence through this multi-stage training program was verified by a practical examination conducted by the physicians conducting the study. The peer tutors had to demonstrate all the practical, theoretical and didactic skills required for the course in this “trial session.

### Assessment instruments

#### Evaluation questionnaire

Participants evaluated the course after completion using a written questionnaire with a 7-point Likert scale (1 = strongly agree; 7 = strongly disagree). The topics “expectations and needs”, “satisfaction with course concept/structure”, “satisfaction with teaching methods/materials/media”, “subjective competency assessment”, and “tutor evaluation” were assessed.

#### Ultrasound exams

To objectively measure ultrasound competency acquisition through the course, the modified OSCE examination questionnaires published by Hofer et al. [34] were applied in paper form. Tutors received didactics training in advance to adequately administer this examination format. In an expert consensus, fixed combinations of the OSCE examination were previously defined with regard to the degree of difficulty of exams. Therefore, in order not to overload the time frame, 2 out of 12 exams were performed per each participant. Each participant was to pass both examination scenarios independent of the course format, with the same combinations of scenarios being tested. Per examination, 50 assessment units could be achieved. In each examination, in addition to practical competences (approx. 80% of the examination = 40 assessment units), theoretical competencies were also measured in the form of questions (approx. 20% of the examination = 10 assessment units). The ratio of each subtype (right kidney, left kidney, inferior vena cava, portal vein, gall bladder, left hepatic lobe, right hepatic lobe, spleen, retroperitoneal space in transverse section, retroperitoneal space in sagittal section, urinary bladder) exam format administered was approximately the same in course models A and B.

### Statistical analysis

Data were entered in Microsoft Office Excel sheets. All statistical analyses and graphics were conducted using R studio (RStudio Team. RStudio: Integrated Development for R. 2020) with R 4.0.3 (R Foundation for Statistical Computing. A Language and Environment for Statistical Computing). Binary and categorical baseline parameters are expressed as absolute numbers and percentages. Continuous data are expressed as median and interquartile range (IQR), or as mean and standard deviation (SD). Categorical parameters were compared using Fisher’s exact test and continuous parameters using the Mann–Whitney U test. P-values < 0.05 were considered statistically significant.

## Results

### Participants

Participants were students of the first clinical semester who had passed the first state examination. Students from five different weekly courses conducted in consecutive semesters between the winter semesters 2017/18 and 2019/20 participated in course model A (n = 744) and 144 students from five different weekend courses conducted in the summer semester 2020 in course model B. Table 1 provides an overview of the participant distribution and the respective evaluation and examination in the different course stages. Reasons for dropping the course included illness or personal reasons.

**Table 1:** Distribution of participants and evaluation and examination formats; WiSe = winter semester; SuSe = summer semester

	Registered participants	Completed evaluation sheets*	Participants in examination*	Completed examination sheets*
WiSe 2017/18 (10-week course)	126	102	102	204
SuSe 2018 (10-week course)	126	116	114	220
WiSe 2018/19 (10-week course)	148	126	118	230
SuSe 2019 (10-week course)	164	140	153	290
WiSe 19–20 (10-week course)	180	147	172	324
SuSe 2020 (2-day compact course)	144	141	141	281
Total 10-day course/2-day compact course	744/144	631/141	659/141	1268/281 1549

*Of the students who started the course, some left the course at different stages and thus did not participate in the final (voluntary) evaluation and examination

### Self-assessment and course evaluation

Table 2 shows the results of the subjective course evaluations and attitudes toward sonography training. Except for the topic complex “satisfaction with the amount of time for course delivery” (10-week course: mean = 2.17, 2-day compact course: mean = 3.32), all topic complexes were rated with mean scale points between 1.11 and 2.04 in both groups. Significant differences between the groups were identified for the topics “satisfaction with the amount of time for course delivery”, “practical skills of tutors”, and “didactic skills of tutors”. For the topic complex “satisfaction with the amount of time for course delivery”, the 10-week course group A indicated significantly higher satisfaction on average. In contrast, the topic complexes “practical skills of tutors” and “didactic skills tutors” were rated significantly better by participants in the 2-day course B.

**Table 2:** Subjective evaluation of course model by the participants

	10-week course (n = 631)	2-day compact course (n = 141)	p-value
	mean (SD)	mean (SD)	
Integration of the diagnostic competency sonography into the medical curriculum	1.17 (0.49)	1.11 (0.38)	0.11
Integration of the diagnostic competency sonography into the compulsive course curriculum	1.37 (0.80)	1.26 (0.73)	0.06
Meeting expectations of the course	1.74 (0.78)	1.91 (0.90)	0.05
Clarity of the concept and outline	1.68 (0.82)	1.78 (0.74)	0.05
Comprehensibility and presentation of the learning objectives	1.68 (0.80)	1.57 (0.70)	0.14
Achievement of the learning objectives	1.90 (0.85)	2.04 (0.92)	0.13
Illustration of the learning content through examples	1.63 (0.80)	1.64 (0.71)	0.52
Satisfaction with the educational materials	1.81 (1.02)	1.69 (0.90)	0.52
Satisfaction with course organization	1.83 (1.1)	1.88 (0.95)	0.18
Satisfaction with the amount of time for course delivery	2.17 (1.19)	3.32 (1.68)	**< 0.01**
Practical skills of tutors	1.26 (0.5)	1.16 (0.41)	**0.03**
Didactic skills of tutors	1.24 (0.49)	1.15 (0.39)	**0.02**

Table 3 shows the results of the subjective competency assessments before and after completion of the course for the different course models. For all topic complexes, the subjective competency assessment improved after the course was completed, with the greatest average improvement reported in both groups for the topic complex “transducer handling”. For the topic complexes “technical knowledge” and “spatial orientation”, significantly greater improvements were observed for participants of the 10-week course model A.

**Table 3:** Subjective competency levels of the participants before and after course completion

	10-week course (n = 631)			2-day compact course (n = 141)			p-value
	**before**	**after**	**difference**	**before**	**after**	**difference**	
	mean (SD)	mean (SD)	mean (SD)	mean (SD)	mean (SD)	mean (SD)	
Personal expert ultrasound knowledge	4.13 (1.10)	2.43 (0.93)	1.71 (1.64)	4.09 (1.51)	2.65 (0.86)	1.43 (1.33)	**< 0.01**
Operation of device	5.51 (1.80)	2.61 (1.17)	2.92 (1.97)	5.68 (1.68)	2.62 (0.96)	3.05 (1.61)	0.96
Handling of sonic transducer	5.16 (2.01)	2.06 (1.10)	3.10 (2.27)	5.64 (1.69)	2.26 (0,84)	3.37 (1.59)	0.72
Spatial orientation	4.52 (1.84)	2.27 (1,09)	2.27 (1. 97)	4.35 (1.56)	2.55 (0,91)	1.78 (1.46)	**< 0.01**
Sono-anatomy	4.82 (1.77)	2.22 (1.05)	2.61 (1.92)	5.09 (1.54)	2.41 (0.89)	2.67 (1.44)	0.85
Organ presentation	4.87 (1.82)	2.20 (1.03)	2.67 (1.99)	5.30 (1.62)	2.40 (0.85)	2.91 (1.56)	0.36
Organ evaluation	5.33 (1.79)	2.41 (1.12)	2.93 (2.01)	5.76 (1.57)	2.58 (0.91)	3.18 (1.49)	0.45
Patient management	4.56 (1.96)	2.06 (1.05)	2.51 (2.13)	4.80 (1.70)	2.19 (0.93)	2.60 (1.63)	0.94

### Ultrasound exams

**Fig. 2:**
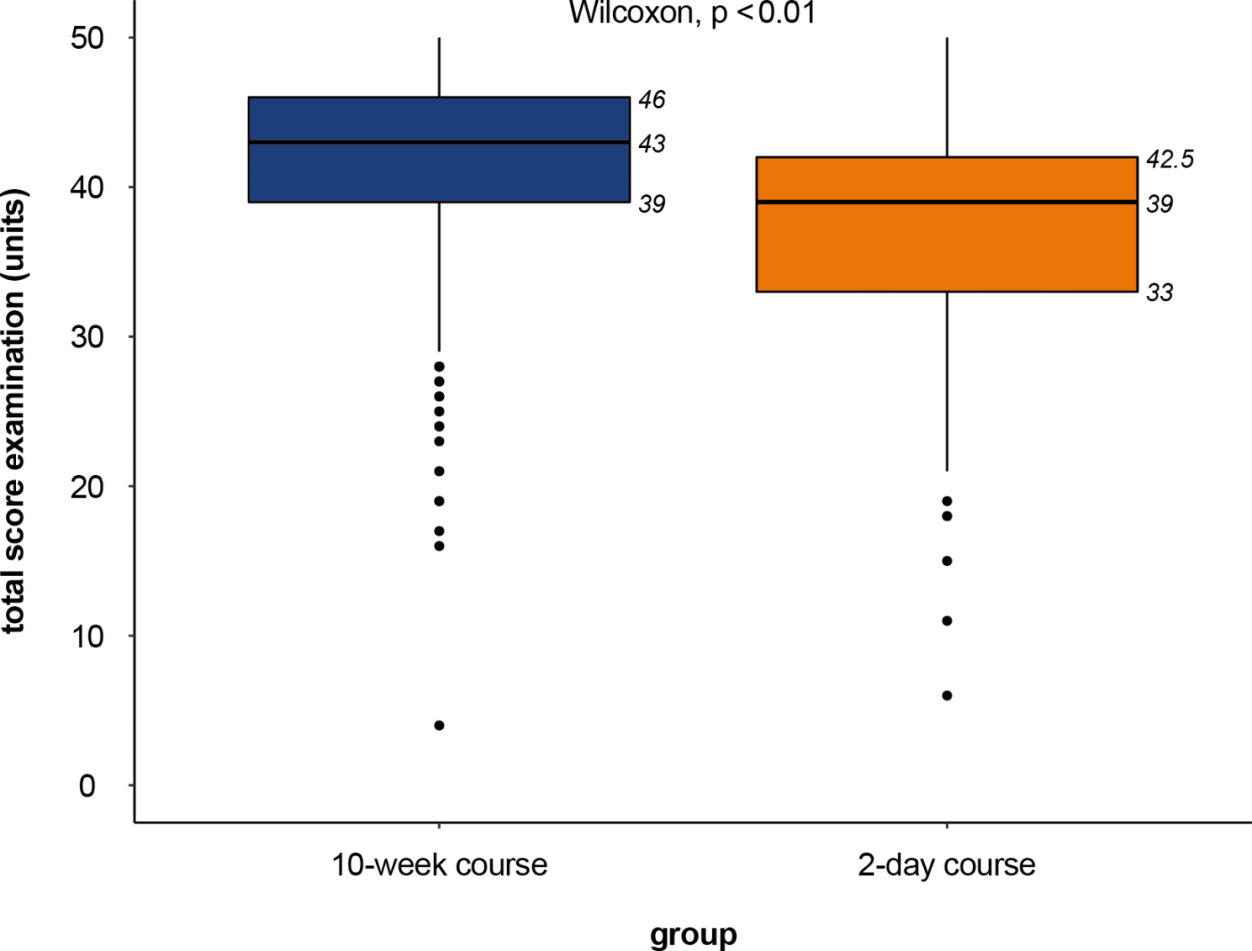
Ultrasound exam scores of participants in the 10-week (blue) and 2-day compact (orange) course models

Mean total scores for the ultrasound exams are presented in Fig. 2. Participants in the 10-week course scored significantly higher on the ultrasound exams than participants in the 2-day compact course (median = 43.0 points per station [IQR 39–46] vs. median = 39 [IQR 33–42.5]; p < 0.01) (Fig. 2).

**Fig. 3:**
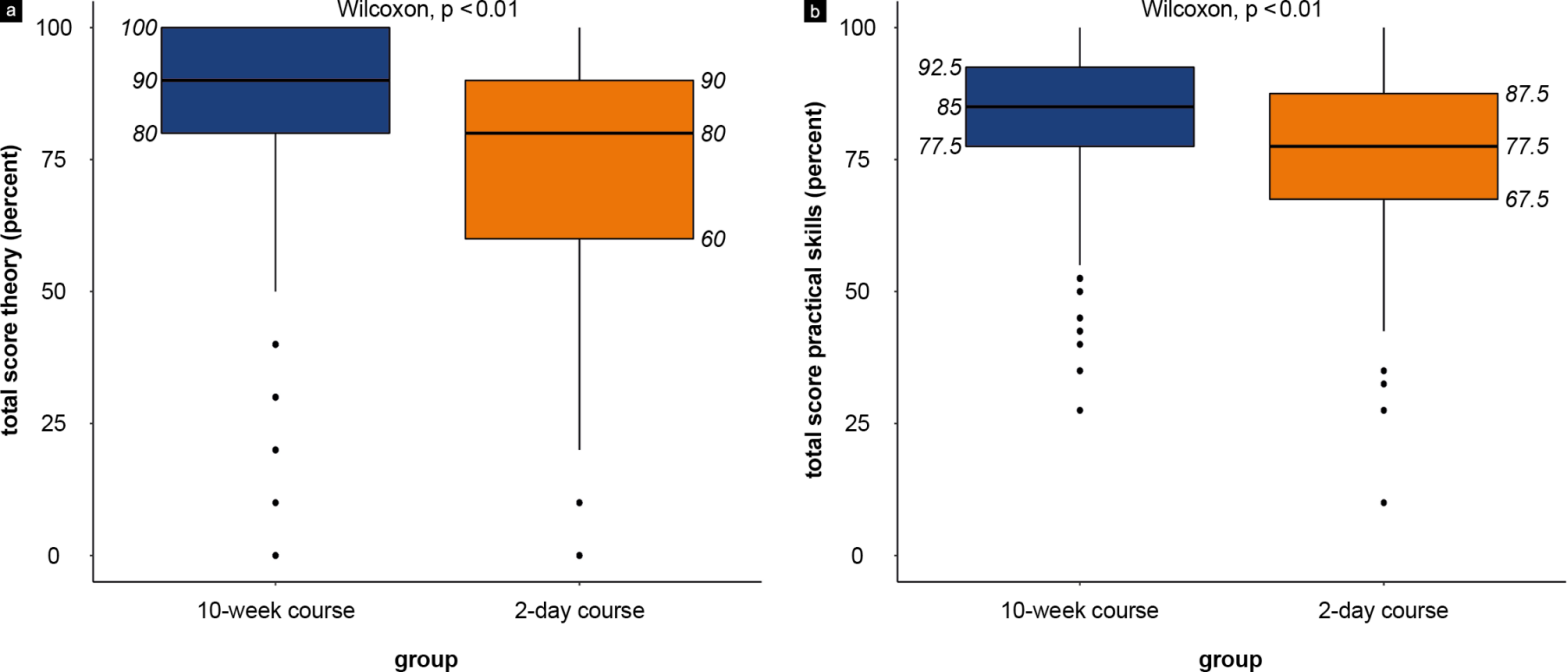
Theoretical (**a**) and practical (**b**) exam results of participants in the 10-week (blue) and 2-day compact (orange) course models in percent

Separate evaluation of theoretical and practical exam results confirmed a consistently higher performance of participants of the 10-week course (Fig. 3). This trend can also be confirmed by looking at the individual subgroups (semesters) for both theoretical and practical ultrasound skills (Supplement Fig. 1).

## Discussion

To the best of our knowledge, this is the first prospective observational study conducted at a university hospital to evaluate and compare different ultrasonography training formats across several semesters. The results demonstrate that the implementation of sonography training in an early clinical semester at a university hospital is feasible. Both subjective perceptions of participants and objective measures of skills showed that didactically designed peer-tutor supported ultrasound education models can build ultrasound competencies. Teaching models delivering the content over several weeks lead to a higher competence level in student sonography training than compact block courses that deliver the content within a few days.

### Discussion of subjective results

The existing high demand for the implementation and integration of sonography course models into the medical curriculum has been addressed in international studies and reviews [5–8, 15]. Our results confirm that students, regardless of the course model attended, would like to see ultrasound-specific competencies taught in their undergraduate or compulsory courses. Because such course models are not available yet in all medical schools, our results provide potential approaches for future course and curriculum designs.

The results of the subjective evaluation show that both training formats were generally accepted by the participants and that the expectations of the course were fulfilled. Irrespective of the course format, the defined course content, including teaching objectives, teaching media and script, as well as the trained peer tutors, was successfully implemented. The very positive evaluation of the tutors illustrated the central importance of the peer-assisted learning approach for the implementation of ultrasound training formats [18]. The significantly better evaluation of the tutors in the 2-day compact course could be explained by the more intensive social contacts in the context of the block courses.

Comparable studies have shown that 90 to 120 min hands-on sessions are favored by participants to convey individual topic complexes (such as aorta, kidney, pelvic organs, FAST) [26–29, 35–37]. Our data confirm these observations, with the significantly lower rated “amount of time for course delivery” in the 2-day compact course group standing out. This could be explained by the large amount of total course material delivered within two consecutive days, which was perceived as too demanding for the participants, or by a decreasing concentration span. In this context, the preparation time as an essential factor for an optimal competency gain in such a compact course format also plays an important role.

A subjective increase in sonography competency was observed both in previous studies as well as in our study, regardless of the course format and duration of the course [15, 28, 29, 38, 39]. Graduates of both course models showed the greatest subjective competency gain in the area “organ assessment”, closely followed by “handling of sonic transducer” and “operation of device”. With regard to the theoretical competency assessment, a higher increase was recorded in the 2-day compact course format group relating to “expert knowledge”. This could be explained by the subjectively perceived steeper learning curve within the compact course format.

### Discussion of objective results

A gain in sonography competency after completion of ultrasound training formats has been observed in numerous studies [26–29]. Overall, the objective examination results obtained in our study suggest that multi-week sonography course models with sequential delivery of the modules lead to a better competence level. Possible reasons could be the lack of continuous small-step preparation and follow-up. In this context, the so-called “spacing effect” could provide possible explanations [40, 41]. In addition, the practical training outside of the course times, which was not offered during the 2-day compact course, could have contributed to a deeper and repeated conveyance of the course contents. In addition to the option of free practice time [15, 37], the consolidation of acquired skills in clinical clerkships and internships [28, 29, 35] is an essential part to retain the knowledge in the long term. Compact course models may be used as a “kick-off” to build up competency.

Because the participants of the 2-day compact course reached a good competency level, this training format could prove particularly useful in pandemic situations [38, 42]. In addition, such short-day compact courses can serve as a useful implementation option for institutions that have not yet developed an ultrasound curriculum for basic medical training. Bundling the teaching time in this format is also attractive from the perspective of the teaching staff, which could improve the quality of teaching in parallel with the time spent in the clinic [21].

Confounding factors or alternative explanations for the observed differences between the course models in our study could be factors such as student characteristics, teaching methods (e.g. video based training), or differences in exam difficulty that may have significantly influenced the results. Our study design does not allow further investigation of the influence of these factors and should be further investigated in future studies.

### Optimizations and future perspectives

Basic ultrasound training implemented in the early clinical stages of medical school can provide the foundation for a longitudinal gain in sonography competency that may be extended and intensified by further training options such as clinical clerkships and clinical coursework. In addition, this could facilitate the transition from medical school into clinical practice and thereby pose a significant advantage for patient care [22, 43, 44]. The results of our study regarding competency development in abdominal sonography should not only contribute to optimizing future curriculum development in this field, but could then also be transferred to other specialty specific ultrasound education programs in fields such as echocardiography, musculoscelettal or head and neck ultrasound. Future multidisciplinary research should be conducted to evaluate which course formats are most fitting to match individual requirements of subspecialty specific ultrasound courses and to thereby verify the transferability of this study. Follow-up studies should evaluate the knowledge or practical skills sustainably acquired until entry into professional life [15, 45, 46]. This could provide conclusions on the optimal design of an ultrasound curriculum within medical school.

For a more effective design of the course preparation time, digital teaching media should be increasingly used in addition to a script in the future as part of a “blended learning approach” [15, 16, 24, 47, 48]. This could possibly lead to a better acquisition of competencies even in the compact course format with a larger scope of topics. The development and use of an appropriate teaching platform is necessary to successfully implement such a blended learning approach.

### Limitations

The preparation time for the respective course model was not assessed in this study and hence could not be correlated with the competence level. The 10-week course group was offered “free practice slots” for independent practice in the Skills Lab. The extent to which this option was used was also not analyzed. In addition, the 10-week course group included students from five different semesters, whereas the students in the 2-day course group completed the course in the same semester. Another limitation is that there was no randomization. The course models could be chosen voluntarily by the participants. The statistical analysis of the ultrasound exam results was only possible for the total group of students because the number of participants of the individual examinations was too small for a subgroup analysis. Furthermore, there was no pre-test before the start of the course to check prior experience. Because the subjective evaluation was anonymous, subjective and objective results could not be correlated. Topic of further discussion should be the influence of each participant preparation time, which is considered a possible influence on the outcome of the practical exam. Irrespective of the course format, a prolonged preparation time could have increased the exam performance.

## Conclusion

Overall, both compact course models such as the 2-day compact course described in this study and training formats lasting several weeks such as the 10-week course are suitable for reaching ultrasound-specific competencies. This can be confirmed not only by the positive subjective evaluations of the participants of the course concepts, the teaching materials, and the tutors, but also by the objectively measurable achievement of competencies in ultrasound exams. The results of this study indicate that multi-week, sequential sonography course models lead to a better ultrasound-specific competency level of students. The aim of further investigations should be to analyze the long-term retention of skills obtained as part of ultrasound training courses in medical school.

## Electronic supplementary material

Below is the link to the electronic supplementary material.

Supplementary Material 1

Supplementary Material 2

Supplementary Material 3

## Data Availability

Data cannot be shared publicly because of institutional and national data policy restrictions imposed by the Ethics committee since the data contain potentially identifying study participants’ information. Data are available upon request from the Johannes Gutenberg University Mainz Medical Center (contact via weimer@uni-mainz.de) for researchers who meet the criteria for access to confidential data (please provide the manuscript title with your enquiry).
