# Supplementary material for: Undergraduate ultrasound training: prospective comparison of two different peer assisted course models on national standards
Source: BMC Med Educ. 2023 Jul 17;23:513. doi: 10.1186/s12909-023-04511-x (PMC10353150; doi:10.1186/s12909-023-04511-x)
Supplement: Supplementary file 1 — Supplementary Material 1 [file 12909_2023_4511_MOESM1_ESM.docx]

**Supplementary Table 1** Learning objectives of the abdominal sonography modules

| Module | Learning objectives | |
| --- | --- | --- |
| 0  Basics | Device operation, **transducer posture patient positioning** **documentation in two layers,** basic ultrasound physics, artefacts, screen orientation, transducer types, ultrasound terminology, essential keyboard functions, ultrasound limitations | |
|  | **Assessed normal findings (image acquisition and identification in the sagittal and transverse plane)** | **Assessed pathologies** |
| 1 + 2a  Vessels | Abdominal aorta, coeliac trunk, superior mesenterial artery, renal arteries, left and right common iliac arteries, inferior vena cava, hepatic veins, splenic vein, portal vein, renal veins | Aortic plaque, aortic aneurysm, aortic dissection, vena cava inferior congestion, benign and malignant lymph nodes |
| 2b  Pancreas | Pancreatic head and body, pancreatic duct, uncinate process, pancreatic tail | Acute and chronic pancreatitis, concretion in ductus pancreaticus, pancreatic lipomatosis, pancreatic carcinoma, congested ductus pancreaticus |
| 3  Portal area of liver, biliary tract, gallbladder | Ductus hepatocholedochus, proper hepatic artery, portal vein, intrahepatic bile ducts, gallbladder pre- and postprandial | Portal vein dilatation, portal vein thrombosis, cholestasis, tumor in biliary duct, biliary calculus, gallstone, cholecystitis, sludge and hydrops, cholesterol polyps |
| 4/  Liver | Hepatic vein star, portal vein plane, intrahepatic bile ducts, liver segments | Diffuse liver lesions (hepatic cirrhosis, Steatosis hepatis), benign and malign focal lesions, Intrahepatic cholestasis |
| 5a/  Kidneys | longitudinal and transverse organ axis, kidney width and length, pyelon/parenchyma ratio, psoas muscle as lead structure “glide sign”, hepatorenal pouch of Morrison, splenorenal pouch of Koller | Form variants, angiomyolipoma, chronic renal failure, renal carcinoma, nephrolithiasis, urinary stasis, pyelonephritis, polycystic kidney |
| 5b  Spleen | Longitudinal and transverse organ axis with measurements, splenic vein, pancreatic tail, volume determination | Splenomegaly with collateral, Splen accessorius, splenic infarction, splenic calcification, splenic cysts, splenic rupture, malign focal lesions |
| 6  Pelvic organs | Urinary bladder including volume determination, urinary bladder jet with color doppler, prostate including measurement, seminal vesicles, uterus including measurement, ovaries, rectum, rectovesical pouch, Douglas pouch, common iliac arteries and veins | Urinary bladder sludge and coagulum, urinary retention, residual urine, chronic cystitis, urinary bladder carcinoma, ascites/free fluid Douglas-room, prostate hyperplasia, ovarian cyst, uterine myoma, intrauterine device |
