# Supplementary material for: Undergraduate ultrasound training: prospective comparison of two different peer assisted course models on national standards
Source: BMC Med Educ. 2023 Jul 17;23:513. doi: 10.1186/s12909-023-04511-x (PMC10353150; doi:10.1186/s12909-023-04511-x)
Supplement: Supplementary file 2 — Supplementary Material 2 [file 12909_2023_4511_MOESM2_ESM.docx]

**Supplementary Table 2** Detailed comparison of course models A (10-week course) and B (2-day compact course), TU = teaching unit

| Model | A: 10-week course | | B: 2-day compact course |
| --- | --- | --- | --- |
| Participants | Students in the first clinical semester after completion of the first state examination | | |
| Before the course | Introductory lecture with course information and basics of ultrasound diagnostics **(2 TU)** | | Distribution of videos with course information and basics of ultrasound diagnostics by mail **(2 TU)** |
| Preparation | Script | | |
| Duration + TU | 10 weeks with **18 TU** (2 TU/course day/week) + final exam (**2 TU**) | | 2 days with **18 TU +** final exam (**2 TU**) |
| Teachers | Peer tutors who had completed a standardized training curriculum under medical supervision (approx. 100 TU) | | |
| Devices | Philips HD 5, Philips Clearvue, Philips CX 50, General Electric LOGIQ F8 | | |
| Location | Examination rooms of the Rudolf Frey Learning Clinic + lecture hall of the university | | |
| Classroom phase | - Theory review and pathology discussion in small groups - Practice with 5 participants per tutor - Absence in one session allowed - 8 written quizzes with 25 points each | | - Plenary theory/pathology discussion in video format - Practice with 3 participants per tutor - No absence allowed - Final written quiz with 30 multiple choice questions |
| Examination | 2 ultrasound exams with 50 assessment units each | | |
| Instructional material | Learning charts with standard sections, standard values, pathologies, and examination procedures | | |
| Contents | 6 identical modules with focus on abdominal sonography | | |
|  | 6 modules on abdominal sonography + 3 excursion modules of 2 TU (90 min):  - chronological completion of the modules 1 to 6  - 3 excursion modules (e-FAST, head-neck glands, vessels and leg veins)  - 25 min theory per module (test + pathology/theory discussion)  - 65 min practice on the device per module  - free practice time in the Skills Lab offered between modules | 6 modules on abdominal sonography + 1 excursion module and 2 revision modules:  **Day 1:**  60 min *Theory* videos modules 1+2  80 min *Practice* modules 1+2 and 80 min *pathology* modules 1+2  30 min *Theory* videos module 3  60 min *Practice* module 3 + 60 min *pathology* module 3  30 min *Theory* videos modules 5 + 6  60 min *Practice* modules 5 + 6 and 60 min *pathology* modules 5+6  **Day 2:**  30 min *Theory* videos module 4  90 min *Practice* module 4 + refresher module  90 min *Pathology* module 4  60 min *Theory* integrated examination day  60 min *Praxis* refresher modules | |
| Theory/practice | 90 + 225 min* / 585 min (groups of 5)  Practice per participant: 117 min | | 90 + 500 min /350 min (groups of 3)  Practice per participant: 116 min |
| After course | Poster with normal and pathological observations + free practice time in the Skills Lab | | |

*Additionally, participants of this group had the voluntary opportunity to listen to a weekly lecture (450 min in total)
