# Supplementary material for: Undergraduate ultrasound training: prospective comparison of two different peer assisted course models on national standards
Source: BMC Med Educ. 2023 Jul 17;23:513. doi: 10.1186/s12909-023-04511-x (PMC10353150; doi:10.1186/s12909-023-04511-x)

**Supplementary figure 1:** Theoretical (a) and practical (b) exam results of participants in the 10-week (blue) and 2-day compact (orange) course model shown by semester. WiSe: winter semester, SuSe: summer semester


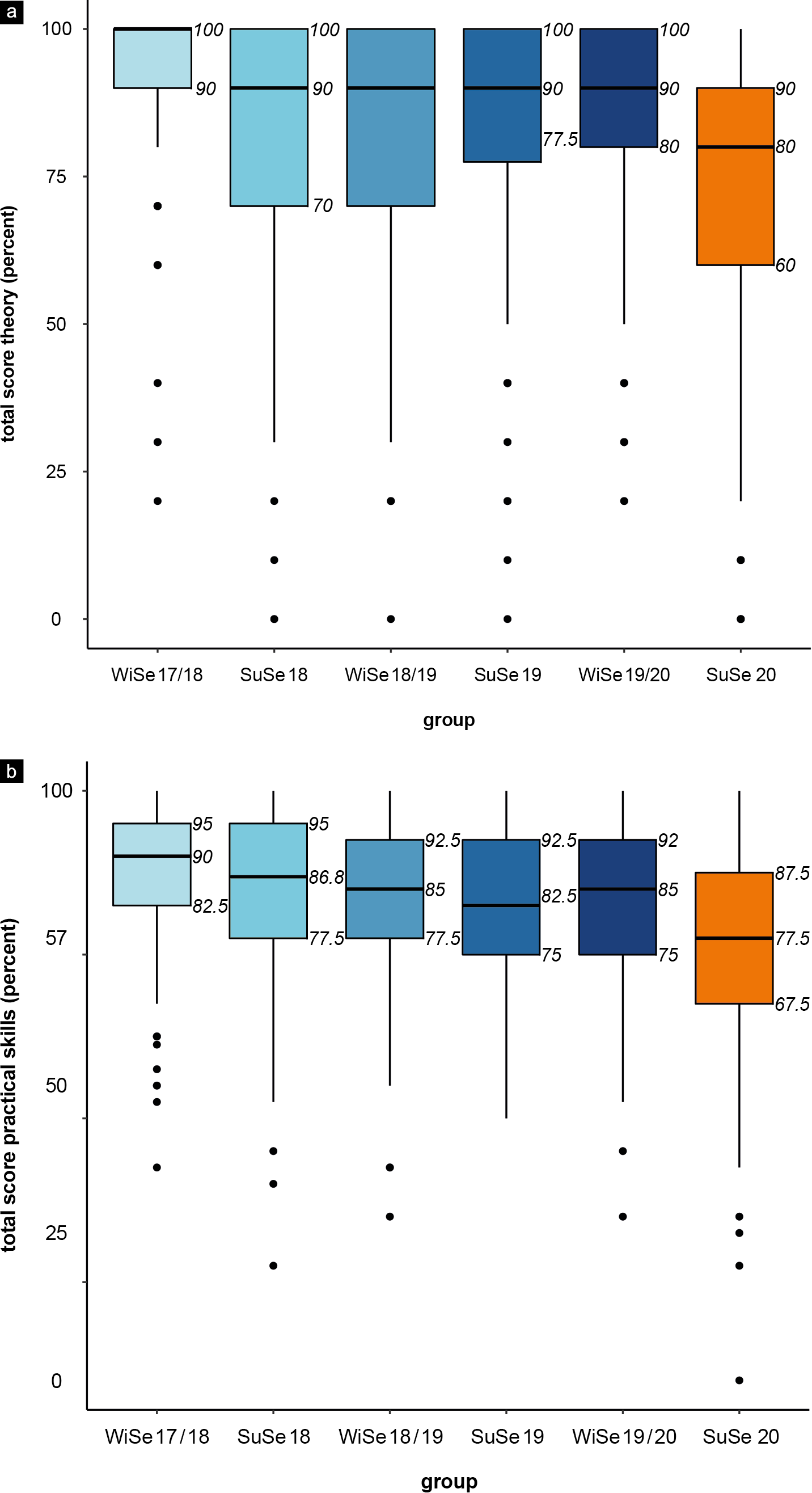

Supplement: Supplementary file 3 — Supplementary Material 3 [file 12909_2023_4511_MOESM3_ESM.docx]
